# Fast nonlinear model predictive planner and control for an unmanned ground vehicle in the presence of disturbances and dynamic obstacles

**DOI:** 10.1038/s41598-022-16226-y

**Published:** 2022-07-15

**Authors:** Subhan Khan, Jose Guivant

**Affiliations:** School of Mechanical and Manufacturing Engineering, University of New South Wales (UNSW), Sydney, 2052 Australia

**Keywords:** Electrical and electronic engineering, Mechanical engineering

## Abstract

This paper presents a solution for the tracking control problem, for an unmanned ground vehicle (UGV), under the presence of skid-slip and external disturbances in an environment with static and moving obstacles. To achieve the proposed task, we have used a path-planner which is based on fast nonlinear model predictive control (NMPC); the planner generates feasible trajectories for the kinematic and dynamic controllers to drive the vehicle safely to the goal location. Additionally, the NMPC deals with dynamic and static obstacles in the environment. A kinematic controller (KC) is designed using evolutionary programming (EP), which tunes the gains of the KC. The velocity commands, generated by KC, are then fed to a dynamic controller, which jointly operates with a nonlinear disturbance observer (NDO) to prevent the effects of perturbations. Furthermore, pseudo priority queues (PPQ) based Dijkstra algorithm is combined with NMPC to propose optimal path to perform map-based practical simulation. Finally, simulation based experiments are performed to verify the technique. Results suggest that the proposed method can accurately work, in real-time under limited processing resources.

## Introduction

In recent years, unmanned ground vehicles (UGVs) have been applied extensively in agriculture, construction, mining and military operations, etc. The UGVs have lightweight, fast speed, low energy consumption, high maneuverability, convenient drive, and simple mechanism^1–3^. Consequently, these advantages make UGVs suitable for indoor^4,5^ and outdoor^6,7^ applications. However, the ground vehicles can be perturbed due to uncertainties in models, and description of the context of operation^8^, skidding and slipping^9^, noisy sensor’s measurements,^10^, and failures in electromechanical components^11^, making the navigation and control of the platform a challenging task^12,13^.

Motion planning and control are the two crucial factors for a vehicle to complete an assigned task safely and effectively^14^. First, it should navigate in an environment with obstacles. Second, it must satisfy the requirement of reaching a specified goal, constantly dealing with perturbations^15^. Thus, the first requirement implied using a path-planner to plan the vehicle’s motion, while the second requirement can be treated by a controller to control the kinematics and dynamics of the vehicle.

In the past few decades, research on motion planning and control of UGVs has witnessed remarkable development. In particular, trajectory tracking and target posture stabilization problems on kinematic models were investigated^16–19^. Additionally, many researchers developed motion control at dynamic levels (such as torque controllers^20,21^ or voltage controllers^22,23^). Moreover, there are some methods developed on model predictive control (MPC), which considers the constraints (such as^24–26^). Similarly, many path-planners and path-followers were developed to reach the specified final location safely^27–30^. From the perspective of UGVs, some studies consider the presence of disturbances, and uncertainties^31–33^; some others assume pure rolling without skid-slip disturbances^34,35^. In addition, some autonomous system use safe switched tracking^36^ and neural network based techniques^37^. Thus, for practical applications, it is important to consider those disturbances.

Overall, most of the existing techniques mentioned above assume that the UGVs satisfy pure rolling conditions (without skid-slip). However, pure rolling may not always hold due to the terrain, tire dynamics, voltage drive circuitry, etc. Thus, making the control problem challenging for the UGVs in the presence of external disturbances and skid-slip. Nevertheless, on the other hand, it allows solving these challenges with advanced planning and control strategies. In^38^, kinematic models that are explicitly linked to skid-slip are presented, which considers the design from a controller’s perspective. An analysis of the hybrid MPC for stabilization of robot under the presence of wheel slippage is discussed in^39^. In^40^, a nonlinear disturbance observer is used for a self-balancing mobile wheeled mobile robot. Additionally, this work focuses on a robust tracking control problem with unknown disturbances. In^41^, an NDO and extended Kalman filter (EKF) are combined to perform the trajectory tracking control for wheeled mobile robots. First, a kinematic model excluding perturbation and distorted dynamic model is discussed. Then, NDO is used to observe the external disturbances, and EKF is used to estimate the platform’s states. In addition, robust constrained control is developed by observing the disturbances using disturbance observer in^42^.

In terms of path-tracking control problem, an obstacle avoidance mobile robot is introduced in^26^. Firstly, a CasAdi-based^43^ single shooting NMPC path-planner is designed. Secondly, a velocity-based virtual control and saturated torque control are designed to apply control actions to the mobile robotic platform. Finally, an extended state observer (ESO) is designed to estimate the disturbances. This technique only considers a single shooting (SS) optimization problem, which requires a larger prediction horizon to compute the NMPC-based path planner. A higher prediction horizon can be understandable in the presence of a complex control problem. However, in the simulations, only a simple point with one dynamic obstacle is avoided. The processing time of the onboard computer is not considered in the simulations, which can cause an issue in the real experiment with the emphasis on the processing time per iteration of the computer. One way to fix the problem is to introduce the multiple shooting(MS) optimization problems, which takes a small prediction horizon. Alternatively, the proximal averaged Newton-type method of optimal control (PANOC) can be used^24^. In terms of voltage control strategies, an adaptive perturbation rejection technique is proposed in^44^. Although they have not considered skid-slip explicitly, the proposed voltage control strategy can solve the trajectory tracking control problem.

This paper focuses on the tracking control problem of a UGV to safely reach a goal location in the presence of moving and static obstacles, skid-slip, and external disturbances. An NMPC-based path-planner, voltage driving control, kinematic and dynamic control, and NDO are used to stabilize the target posture, while PPQ-Dijkstra is used to generate optimal path for the map-based simulation, which is also used in^45^. The main contributions of this research are: (1) In contrast to^26^, the proposed NMPC-based path planner is designed to propose a suitable trajectory to achieve the goal location and avoid the vehicle from colliding with multiple dynamic and static obstacles. Additionally, the MS optimization problem and voltage control strategy are proposed in this paper, which improves the processing time of a single or multicore CPU. (2) A novel EP tuning method is used to tune the gains of the KC to generate velocity commands to the dynamic controller. A dynamic controller and NDO are then proposed to provide voltage-based control actions to the UGV and compensate for the lumped disturbances, respectively. (3) An investigation is performed to measure the single-core and multi-core CPUs processing time per iteration using the proposed MS-based NMPC with the SS method in^26^. In this paper, the proposed control scheme is designed to cope with the dynamic environment. To verify the working of the proposed scheme, extensive simulations have been carried out to show the vehicle’s behavior in the presence of disturbances in a realistic framework.

## Problem formulation

In this paper we use dynamic and kinematic models which consider disturbances, including skidding and slippage; those models are the following ones^46,47^

1 $$\begin{aligned}&{\dot{q}} = S({q})\cdot (\Omega - \eta ) + \rho (q, \mu ) \end{aligned}$$

2 $$\begin{aligned}&M\cdot ({\dot{\Omega }} - {\dot{\eta }}) + H_1(q,{\dot{q}})\cdot (\Omega - \eta ) + H_2(q)\cdot {\dot{\rho }}(q,\mu ) + H_3(q)\cdot \rho (q,\mu ) = B\cdot ((\tau - \tau _d) \end{aligned}$$

with,

$$\begin{aligned} \begin{aligned}{}&q = [x,\,y,\,\theta ]^T \in {\mathbb {R}}^{3}, \Omega = [v,\,\omega ]^T, \eta = [\eta _1, \, \eta _2]^T \in {\mathbb {R}}^{2}, \tau _d = [\tau _{d_{1}},\, \tau _{d_{2}}]^T \in {\mathbb {R}}^{2}, \eta _1 = \frac{r}{2}(\eta _r + \eta _l), \eta _2 = \frac{r}{2b}(\eta _r - \eta _l),\\&m = m_c + m_w, H_1(X,{\dot{q}}) = \begin{bmatrix} 0 &{} -m_cd{\dot{\theta }} \\ m_cd{\dot{\theta }} &{} 0 \end{bmatrix}, H_2(q) = \begin{bmatrix} m + \frac{2J}{r^2}\cos \theta &{} (m + \frac{2J}{r^2})\sin \theta &{} 0 \\ -m_cd\sin \theta &{} m_cd\cos \theta &{} J + \frac{2b^2J}{r^2} \end{bmatrix}, \\&H_3(q) = \begin{bmatrix} \frac{-2J{\dot{\theta }}\sin \theta }{r^2} &{} \frac{2J{\dot{\theta }}\cos \theta }{r^2} &{} -m_cd{\dot{\theta }}\\ 0 &{} 0 &{} 0 \end{bmatrix}, S(q) = \begin{bmatrix} \cos \theta &{} \sin \theta &{} 0 \\ 0 &{} 0 &{} 1\end{bmatrix}^T, \rho (q, \mu ) = \begin{bmatrix} -\mu \sin \theta \\ \mu \cos \theta \\ 0 \end{bmatrix}^T, \\ {}&M = \begin{bmatrix} m &{} 0 \\ 0 &{} J - md^2 \end{bmatrix}, \quad \tau = [\tau _1, \tau _2]^T, \in {\mathbb {R}}^{2},\quad H = [H_1(q,{\dot{q}}), H_2(q),H_3(q)]^T \end{aligned} \end{aligned}$$

**Figure 1:**
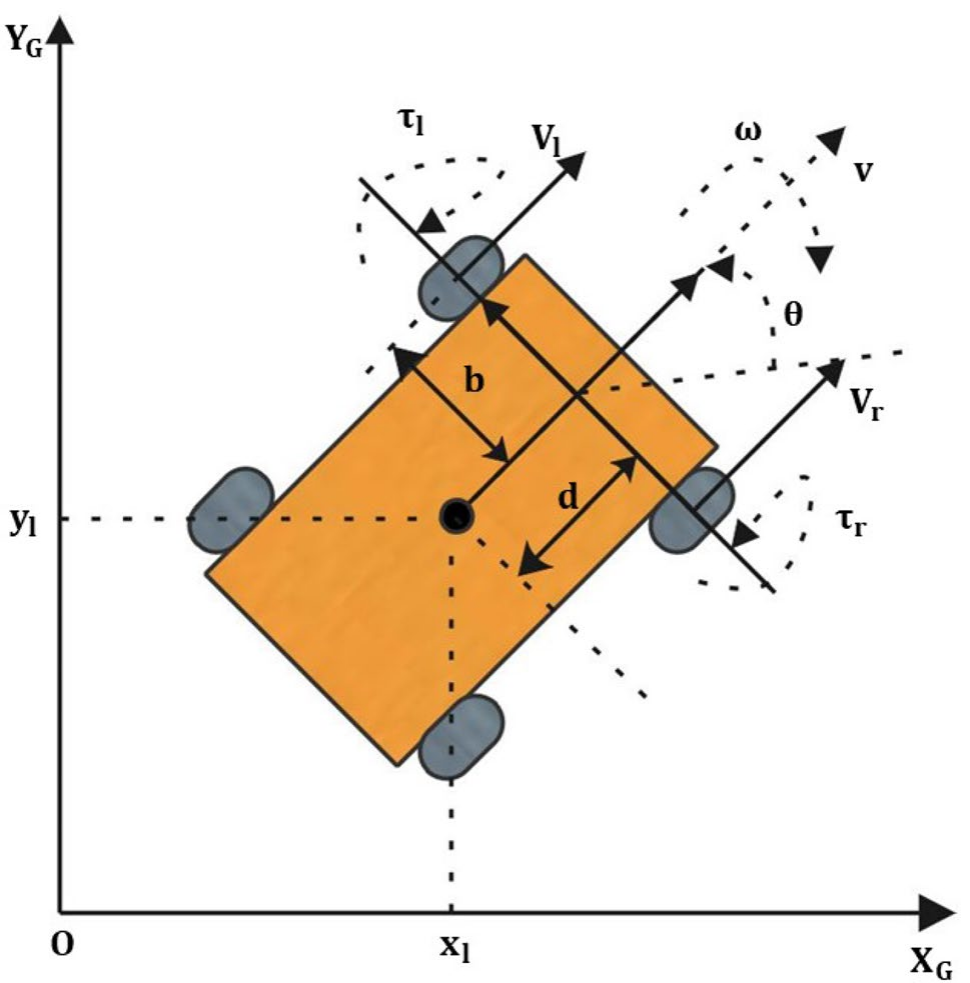
UGV configuration.

Consider the UGV represented in Fig. 1 is driven by two DC motors attached on each side with same mechanical properties with $N > 1$. An expression for the angular velocities of motor could have the following expression:

$$\begin{aligned} \omega _l&= \frac{v + b\cdot \omega }{r}, \qquad \omega _r = \frac{v - b\cdot \omega }{r} \end{aligned}$$

For simplicity, we have assumed that the torque $\tau$ generated by the motors are explicitly depended on the armature current

3 $$\begin{aligned} \tau&= N\cdot K_t \cdot i_a, \end{aligned}$$

where $K_t$ is the torque constant. Now, we can obtain the armature voltage as follows

4 $$\begin{aligned} V_u&= L_a \frac{di_a}{dt} + R_a i_a + N \cdot K_f \cdot \omega _a \end{aligned}$$

where $V_u = [V_r,\,V_l]^T$. Now, we can convert our torque dynamics into the voltage driving circuit by using the above expression as follows:

5 $$\begin{aligned} \begin{bmatrix} V_l \\ V_r \end{bmatrix}&= \frac{L_a}{N K_t} \begin{bmatrix} {\dot{\tau }}_l \\ {\dot{\tau }}_r \end{bmatrix} + \frac{R}{N K_t} \begin{bmatrix} \tau _l \\ \tau _r \end{bmatrix} - \frac{K_f}{r} \begin{bmatrix} -N & -N b \\ -N &{} N b \end{bmatrix} \begin{bmatrix} v \\ \omega \end{bmatrix} \end{aligned}$$

## Methods

**Figure 2:**
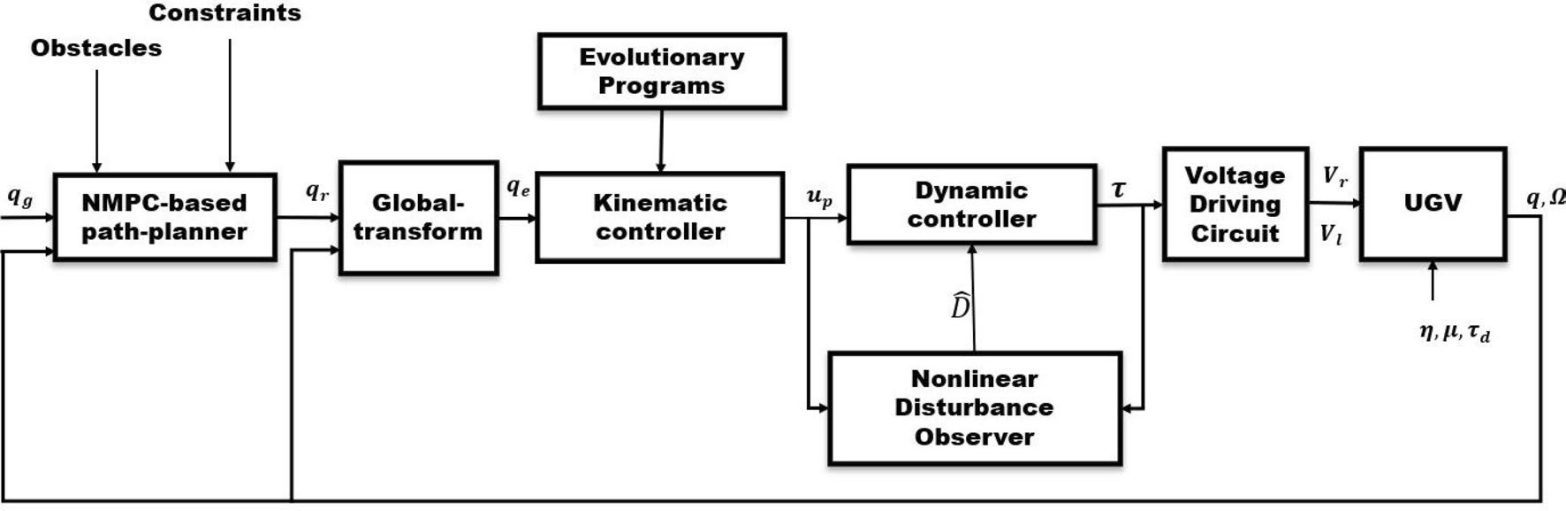
Proposed planner and control structure diagram for the UGV.

In this Section, first, an NMPC-based path planner is to generate reference trajectory to reach the current destination pose, additionally satisfying the collision avoidance constrain. Second, an EP-based gain tuning method is designed to generate KC-based velocity commands. Third, a dynamic control is designed with the NDO to mitigate the effects of disturbances and treat the skid-slip effect. Finally, the stability analysis of the proposed controller is discussed. Figure 2 illustrates the overall close-loop structure of the proposed technique.

### NMPC-based path planner

we will consider NMPC-based path planner to generate a reference trajectory for the UGV from a starting pose *q*(0). In particular, the the presences of static and dynamic objects are considered in the environment of the UGV. For the reference posture of the UGV, we consider that the platform operates free of skid-slip, which corresponds to the assumption of pure rolling. Thus, by using the pure rolling, the kinematics and dynamics of the UGV can be expressed as^48^

6 $$\begin{aligned} {\dot{q}}_r&= S(q_r)\cdot \Omega _r,\qquad {\dot{\Omega }}_r = a_r \end{aligned}$$

where $a_r = [{\dot{v}}\,, {\dot{\omega }}]^T$ and $\Omega _r = [v_r,\, \omega _r]^T$. Until now, we have taken we have modelled the platform by a continuous time model. as a continuous-time system. However, it is essential for a computer to generate discrete signals. Therefore, by using Euler’s approximation with $T_s$, we can approximate our nonlinear continuous-time system by a discrete-time one, as follows

7 $$\begin{aligned} q_r(k+1)&= q_r(k) + T_s\cdot {\dot{q}}_r(k),\qquad \Omega _r(k+1) = \Omega _r(k) + T_s\cdot a_r(k) \end{aligned}$$

The discrete indexing $k \in \Lambda$, while $\Lambda$ is a set of positive natural numbers.

In terms of collision avoidance, the UGV must move with some restrictions. Therefore, we have set constraints on the platform’s physical position as follows:

8 $$\begin{aligned} g^{i}_{o}(q_r(k))&= \sqrt{(x(k) - x^{i}_{o}(k))^2 + (y(k) - y^{i}_{o}(k))^2} - (r_c + r^{i}_{o}) \end{aligned}$$

where obstacles are $i = 1,2,\dots ,I$; *I* represent number of currently known obstacles. Additionally, constraints on the velocities and accelerations are considered, as follows:

9 $$\begin{aligned} v_{r_{min}} \le v_{r}(k) \le v_{r_{max}}, \qquad \omega _{r_{min}} \le \omega _{r}(k) \le \omega _{r_{max}},\qquad {\dot{v}}_{r_{min}} \le {\dot{v}}_{r}(k) \le {\dot{v}}_{r_{max}}, \qquad {\dot{\omega }}_{r_{min}} \le {\dot{\omega }}_{r}(k) \le {\dot{\omega }}_{r_{max}}. \end{aligned}$$

Now, we can compute the cost-function for our NMPC-based path planner as follows:

10 $$\begin{aligned} \underset{a_r}{\arg \min } \quad J(a_r(k + j))= C_T(q_{N_{p}},\Omega _{N_{p}}) +&\sum _{j = 0}^{{N_{p}} - 1} C_S(q_r(k+j),\Omega _r(k+j),a_r(k+j)) \end{aligned}$$

subject to following constraints

11 $$\begin{aligned} \begin{aligned} q_r(k + j)&= f_1(q_r(k + j - 1),\Omega _r(k + j - 1)),\qquad \Omega _r(k + i) = f_2(\Omega _r(k + i - 1),a_r(k + j -1)),\\&a_r(k + j - 1) \in U, j\in {\mathbb {N}}_{[0,\, {N_{p}}-1]},\qquad g^{i}_{o}(q_r(k+j)) \le 0, j \in {\mathbb {N}}. \end{aligned} \end{aligned}$$

where $C_T(q_{N_{p}},\Omega _{N_{p}}) = (q_{N_{p}} - q_g)^T\cdot Q_{N_{p}}\cdot (q_{N_{p}} - q_g) + \Omega _{N_{p}}^T \cdot \bar{Q}_{N_{p}} \cdot \Omega _{N_{p}}$, $C_S(q_r,\,\Omega _r,\,a_r) = (q_r - q_g)^T\cdot Q \cdot (q_r - q_g) + \Omega _r^T \cdot \bar{Q} \cdot \Omega _r + a_r \cdot R \cdot a_r^T$, $j = 1, \dots N$, The nonlinear problem proposed in (10) and (11) can be transformed into optimal control problem by using the multiple shooting numerical method proposed by^43^. Alternatively, the single shooting method, as proposed in^26^, can be used. However, it would be computationally more complex.

### Kinematic controller

The evolutionary strategies narrow the degree of freedom in the instantiating of meta-heuristic algorithms. Thus, the main idea here is first to take the error between the reference and actual positions and orientation.

12 $$\begin{aligned} \tilde{q_e}&= q_r - q = \begin{bmatrix} x_e \\ y_e \\ \theta _e \end{bmatrix} = \begin{bmatrix} x_r - x \\ y_r - y \\ \theta _r - \theta \end{bmatrix} \end{aligned}$$

Now, by taking the global transformation mentioned in^49^, we take the following posture error of the real UGV from (12):

13 $$\begin{aligned} q_e&= \begin{bmatrix} e_1 \\ e_2 \\ e_3 \end{bmatrix} = \begin{bmatrix} \cos (\theta ) &{} \sin (\theta ) &{} 0 \\ -sin(\theta ) &{} \cos (\theta ) &{} 0 \\ 0 &{} 0 &{} 1 \end{bmatrix} \cdot \begin{bmatrix} x_e \\ y_e \\ \theta _e \end{bmatrix} \end{aligned}$$

The time derivative of the tracking error of (13) is as follows

14 $$\begin{aligned} \begin{bmatrix} \dot{e_1} \\ \dot{e_2} \\ \dot{e_3} \end{bmatrix}&= v \begin{bmatrix} -1 \\ 0 \\ 0 \end{bmatrix} + \omega \begin{bmatrix} e_2 \\ -e_1 \\ -1 \end{bmatrix} + \begin{bmatrix} v_r\cos (e_3) \\ v_r\sin (e_3)\\ \omega _r \end{bmatrix} \end{aligned}$$

Now, a back-stepping method (as that presented in^49^) is used to get a control signals excluding skid-slip.

15 $$\begin{aligned} u_{c}&= \begin{bmatrix} v_{c} \\ \omega _{c} \end{bmatrix} = \begin{bmatrix} v_r\cos (e_3) + k_x e_1 \\ \omega _r + k_y v_re_2 + k_\theta v_r\sin (e_3) \end{bmatrix} \end{aligned}$$

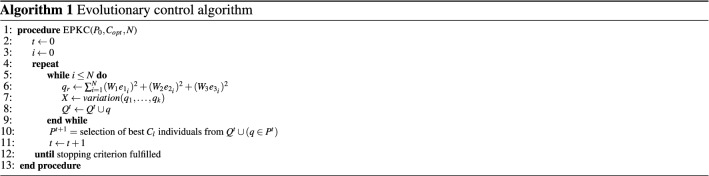


In EP, we only require to know the fitness function of an individual population. Consider *P* as a population of all parameters involved in Eq. (1) and $k^* = [\hat{k_x},\,\hat{k_y},\,\hat{k_\theta }\,] \in {\mathbb {R}}^{3}$. Now, the main idea here is to find sub-optimal scaling factors $\hat{k_x},\,\hat{k_y}$ and $\hat{k_\theta }$. These sub-optimal scaling factors can be achieved by applying the EP searching algorithm shown in Algorithm 1.

16 $$\begin{aligned} u_{P}&= \begin{bmatrix} v_{P} \\ \omega _{P} \end{bmatrix} = \begin{bmatrix} v_r\cos (e_3) + \hat{k_x}e_1 \\ \omega _r + \hat{k_y}v_re_2 + \hat{k_\theta }v_r\sin (e_3) \end{bmatrix} \end{aligned}$$

The error function $u_e = [v_e,\,\omega _e]^T \in {\mathbb {R}}^{2}$ of velocities can also be computed as follows

17 $$\begin{aligned} v_e&= -x_e\sin \theta + \omega y_e\cos \theta + v - v_r\cos \theta _e, \qquad \omega _e = \omega - \omega _r \end{aligned}$$

### Dynamic controller with NDO

The dynamics in Eq. (2) can be transferred into the following

18 $$\begin{aligned} \bar{M}\cdot {\dot{u}}_{P} + F = \bar{B}\cdot \tau \end{aligned}$$

with

$$\begin{aligned} \begin{aligned} \bar{M}&= T^TMT, \bar{B} = T^TB, \quad T = \begin{bmatrix} (x_e\sin \theta - y_e\cos \theta ) &{} 1 \\ 1 &{} 0 \end{bmatrix},\Pi = [v_r\cos \theta _e+\omega _r(x_e\sin \theta -y_e\cos \theta ),\, \omega _r]^T.\\ F&= T^T[M{\dot{T}}(u_e + \Pi ) + MT{\dot{\Pi }} - M{\dot{\eta }} + C_1(\Omega - \eta )] + T^T[C_2{\dot{\rho }} + C_3\rho ] + \bar{B} \cdot \tau _d, \end{aligned} \end{aligned}$$

The parameter *M* is unknown. However, the nominal $M_0$ is known, and taking the velocity tracking error $\zeta = u_e - u_{P}$, we have

19 $$\begin{aligned} {\dot{\zeta }}&= \bar{M_0}^{-1} \bar{B} \cdot \tau + H \cdot \bar{B} \cdot \tau - \bar{M}^{-1} \cdot F - {\dot{u}}_{P} \end{aligned}$$

where $H = \bar{M}^{-1} - \bar{M_0}^{-1}$. In addition, we can take $\Delta \tau = (\tau (k+1) - \tau (k))$ to handle the saturation constraints. This will also gives an intuition that $\dot{u_{P}}$ is related to unknown skid-slip. Therefore the above expression becomes as follows

20 $$\begin{aligned} \begin{aligned} {\dot{\zeta }}&= \bar{M_0}^{-1} \bar{B}\tau + \Delta \tau + H \cdot \bar{B} \cdot \tau - \bar{M}^{-1}F - {\dot{u}}_{P} - (\bar{M_0}^{-1} \bar{B} - I)\Delta \tau \end{aligned} \end{aligned}$$

To accomplish the NDO, we first need to lumped the estimated disturbances as follows

21 $$\begin{aligned} \hat{{\dot{\zeta }}}&= L(q) \cdot (\zeta - {\hat{\zeta }}) \end{aligned}$$

where *L*(*q*) is the observer gain. Now, we can define the auxiliary variable $\hat{D}$ to obtain an improved disturbance observer design as follows:

22 $$\begin{aligned} \hat{{\dot{D}}}&= L(q) \cdot (\bar{M_0}^{-1} \bar{B} \cdot \tau + H \cdot \bar{B} \cdot \tau - \bar{M}^{-1} \cdot F - {\dot{u}}_{P}) - L(q) \cdot \hat{{\dot{\zeta }}}, \qquad {\hat{\zeta }} = \hat{D} + p(u_{P}) \end{aligned}$$

where the function $p(u_p)$ is selected from^50^.

23 $$\begin{aligned} p(u_{P})&= K \cdot u_{P}, \qquad {\dot{p}}({u_{P}}) = K \end{aligned}$$

where K is the constant matrix. Now, by taking the time derivative of (22)

24 $$\begin{aligned} \hat{{\dot{\zeta }}}&= \hat{D} + {\dot{p}}(u_{P})\cdot {\dot{u}}_{P} \end{aligned}$$

By combining the expressions Eqs. (21) and (22)

25 $$\begin{aligned} \hat{{\dot{\zeta }}} - \hat{{\dot{D}}}&= L(q) \cdot \bar{M}{\dot{u}}_{P}, \qquad L(q)\cdot \bar{M}={\dot{p}}(u_{P}) \end{aligned}$$

Thus, we select the observer gain matrix as $L(q) = K\cdot \bar{M}^{-1}$. Now, using Eqs. (19) and (22) the expression can be re-written as follows:

26 $$\begin{aligned} {\dot{\zeta }}&= \bar{M}^{-1}_{0}\cdot \bar{B} \cdot \tau + \Delta \tau + \hat{D} \end{aligned}$$

For the system proposed in (26) the applied torque can be achieved as follows:

27 $$\begin{aligned} \tau&= \bar{M_0}^{-1} \cdot \bar{B} \cdot \left[ -\alpha \cdot \zeta + \beta \cdot K_c - \frac{\beta ^2 \zeta }{2} - \hat{D}\right] \end{aligned}$$

The stability analysis of the observer is performed as per^26^. Therefore, we will discuss the closed loop stability analysis of the controller scheme in the following subsection.

### Stability analysis

**Figure 3:**
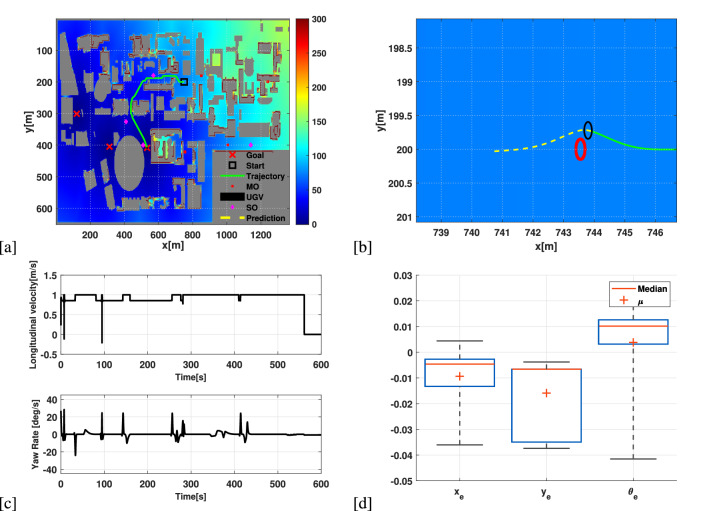
(**a**) Path-tracking of the UGV with a birds eye-view of the UNSW map. (**b**) Collision avoidance of incoming MO as well as the prediction of future state. (**c**) Applied longitudinal and angular velocity of the UGV. (**d**) Tracking error representation of the vehicle’s actual states.

To provide the stability analysis of the closed loop system in Fig. 3, we will first define an important assumption as follows:

#### Assumption 1^49^

Consider a first order continuous differential variable $\alpha _1(t) \in [0, \,\infty )$, which has a limit with $t \rightarrow \infty$. Thus, the second derivative of $\alpha (t)$ does existed and bounded for all $t \in [0,\, \infty )$ such that first derivative holds the condition $\lim _{t \rightarrow \infty }\dot{\alpha _1}(t) = 0$.

Consider the model of UGV Eqs. (1) and (2), the posture control error Eqs. (17) and (20), the NDO Eqs. (21)–(26), and controllers Eqs. (5) and (27). Then a compensated error-inequality as follows

28 $$\begin{aligned} \Vert {e_c}^2\Vert&\ge \frac{H_{max}}{K_{c_{min}} - \Vert \bar{C_m}\Vert } (K_{c_{min}} > \Vert \bar{C_m}\Vert )&\end{aligned}$$

can be refined and we can find error-based asymptotic stability.

while,

29 $$\begin{aligned} \bar{C}_m&= C_1(q,{\dot{q}})(\Omega - \eta ) + C_2(q){\dot{\rho }}(q,\mu ) + C_3(q)\rho (q,\mu )&\end{aligned}$$.

Consider the following Lyapunov function

30 $$\begin{aligned} V_1&= k_1(e_x + l(1 - \cos (e_\theta ))^2 + k_1(e_y - l \sin (e_\theta ))^2 + 2k_3 v_r(1- \cos (e_\theta )) + V_2 \end{aligned}$$

where $V_2 = \frac{e_c^T\bar{M}e_c}{2}$. In addition, the Lyapunov function $V_1 \ge 0$ and $V_1 = 0$ when $e = 0$ and $e_c = 0$. Now, by taking the following derivative

31 $$\begin{aligned} \dot{V_1}&= 2k_1(e_x + l(1 - \cos (e_\theta ))({\dot{e}}_x + l \sin (e_\theta ){\dot{e}}_\theta ) + 2k_1(e_y - l\sin (e_\theta )({\dot{e}}_\theta - l\cos (e_\theta ) + 2k_e v_r \sin (e_\theta ){\dot{e}}_\theta + {\dot{V}}_2 \end{aligned}$$

Let the function of Eq. (20) has an error $e_F = F - \hat{F}$, and controller of Eq. (27), we can differentiate V2 and get

32 $$\begin{aligned} \dot{V_2}&= \frac{1}{2}e_c^T(\dot{\bar{M}}-2\cdot \bar{C_m})\cdot e_c + e_c^T\cdot (-K_c\cdot e_c + e_F)&\end{aligned}$$

It is important to notice here that skew symmetry property makes the first term zero, which allows us to rewrite the $\dot{V_2}$ as

33 $$\begin{aligned} \begin{aligned} \dot{V_2}&= e_c^T (-K_c\cdot e_c + e_F) \le (-K_{c_{min}} \Vert {e_c}^2\Vert + \Vert {C_m}\Vert \Vert {e_c}^2\Vert + \Vert {e_c}\Vert H_{max}) \end{aligned} \end{aligned}$$

where $H_{max}$ is the maximum NDO estimation error. Now, we can substitute the inequality and the derivatives of the velocity error in Eq. (17), we have

34 $$\begin{aligned} \begin{aligned} \dot{V_1}&\le 2\cdot k_x \cdot e_x (v_r\cdot \cos (e_3)-v_c) + (2\cdot k_y \cdot v_r\cdot e_2) + 2\cdot k_\theta v_r \cdot (\omega _r - \omega _c)\cdot \sin (e_3)- K_{c_{min}}|{e_c}\Vert ^2 + \Vert {\bar{H_m}\Vert }\Vert {e_c}^2\Vert + \Vert {e_c}C_{maxc}\Vert \end{aligned} \end{aligned}$$

Now, we will introduce EPKC from Eq. (22), defining $\hat{k_y} = \frac{k_x}{\hat{k_\theta } v_r}$ and substitute in Eq. (32).

35 $$\begin{aligned} \dot{V_1}&\le -2{\hat{k}^2}_{x^{x}} - 2\hat{k} _\theta ^2 \cdot v_r^2 \sin (e_3)^2 + \Vert {e_c}^2\Vert (-K_{c_{min}} + \Vert {C_m}\Vert ) + \Vert {e_c}H_{max}\Vert \end{aligned}$$

In which it can be seen that the first two terms of the above equation are negative, which also guarantees negative $\dot{V_1}$ as long as the following is satisfied

36 $$\begin{aligned} \Vert {e_c}\Vert&\ge \frac{H_{max}}{K_{c_{min}} - \Vert {C_m}\Vert }&\end{aligned}$$

Therefore, we can write-down the velocity error between EPKC and the estimation velocity as

37 $$\begin{aligned} e_c&= \begin{bmatrix} \bar{v}_e\\ {\bar{\omega }}_e \end{bmatrix} = \begin{bmatrix} (v_r\cos (e_3) + \hat{k}_x e_1) - \hat{v}\\ (\omega _r + \hat{k}_y v_r e_y + \hat{k}_\theta ) - {\hat{\omega }} \end{bmatrix}&\end{aligned}$$

The error functions $e_s = [e\,,e_c]^T$ is bounded, therefore, both $\Vert {e}\Vert$ and $\Vert {{\dot{e}}}\Vert$ are bounded. In addition, $\lim _{t \rightarrow \infty }\dot{\dot{V_1}} = 0$ can be obtained from the Assumption 1. Thus, it can be concluded that posture and velocity errors of the UGV are asymptotically stable.

## Results and discussions

In this section, we discuss the simulation-based experiments performed in MATLAB. The physical parameters of UGV are set as follows: $b = 0.67\, \mathrm{m}$, $r = 0.30\,\mathrm{m}$, $d = 0.6\,\mathrm{m}$, $m_c = 50\,\mathrm{kg}$, $m_w = 2\, \mathrm{kg}$, $J = 15\, \mathrm{kg \,m}^{2}$. The skid-slip are considered as $[\eta _1,\,\eta _2,\mu ] = 0.02\cdot [\sin (0.3t),\,1, \,1]$. The external torque-based disturbance $t_d = [0.01\cos (0.5t) 0.01\sin (0.5t)]^T$.

For NMPC gain tuning, we have selected the weighting matrices $Q = diag[5,\,10,\,0.01]$, $\bar{Q} = diag[10,\,10,\, 10]$, and $R=diag[0.05,\,0.05]$. The prediction horizon of ${N_{p}} = 20$ for the simulations related to path-tracking, and ${N_{p}} = 22$, the simulations related to tracking a circle. Additionally, the sample time of $T_s = 0.01\,{s}$ is selected. The input torque is saturated and assumed to be $\tau _{min} = -100 \,\mathrm{Nm}$ and $\tau _{max} = 100\,\mathrm{Nm}$. Finally, voltages on the right and left motors are $V_{r_{min}} = -12 \,{V}$, $V_{r_{max}} = 12\,{V}$, $V_{l_{min}} = -12 \,{V}$ and $V_{l_{max}} = 12\,{V}$, respectively.

### Simulation 1: Path-tracking in a large map using PPQ-Dijkstra

In this path-tracking experiment, the tracking process is combined with a 2 DoF optimal planner (PPQ-Dijkstra), and considered the University of New South Wale’s evacuation map. The map has a size of (1350 m $\times$ 600 m) which consists of the buildings of the campus. The intuition behind the experiment is to avoid moving obstacles (MOs) and static obstacles (SOs) in a large map. For this purpose, three destinations are provided as shown in the Fig. 3a, while UGV able to select for selecting the shortest path from the PPQ-Dijkstra (which can deal with multiple destinations). The color lines in the Fig. 3a represents how expensive it is to reach the goal. For example, red has the highest value of 300 which refers to a higher cost, so it is necessary to follow the vehicle to follow blue color (0 value) in order to follow the lowest cost. The NMPC is looking after the non-holonomic constraints of the vehicle as well as the collision avoidance. Figure 3b represents the UGV avoiding the incoming MO, additionally, providing the prediction of the vehicle’s state.

In terms of the performance of the proposed scheme, Fig. 3c represents the velocity profile applied to the real-platform. Clearly, the chattering in both longitudinal velocity and yaw rate represents the collision avoidance in the environment. As far as tracking errors are concerned, Fig. 3d shows that the actual states of the UGV has a good accuracy.

### Simulation 2: Circle trajectory tracking

For this simulation experiment, we have considered the following expression to form a circle:

38 $$\begin{aligned} \Bigg \{ x_r(t)&= 5sin(0.2 t), \qquad y_r(t) = 5cos(0.2 t) \end{aligned}$$

where $t \in [0,\, 25]$, for the above expression, the initial conditions are aligned with the reference trajectory as $[x,\,y,\,\theta ]=[0,\,,0\,,0]$.The prediction horizon for this experiment is ${N_{p}} = 22$.The input constraints are selected differently for this experiment. We have the following input constraints:

$$\begin{aligned} -1 {m/s}&\le v \le 1 {m/s}, \quad -45 {deg/s} \le \omega \le 45 {deg/s}, \quad -1 {m/s^2} \le a_{r1} \le 1 {m/s^2}, \quad -45 {deg/s^2} \le a_{r2} \le 45 {deg/s^2}. \end{aligned}$$

**Figure 4:**
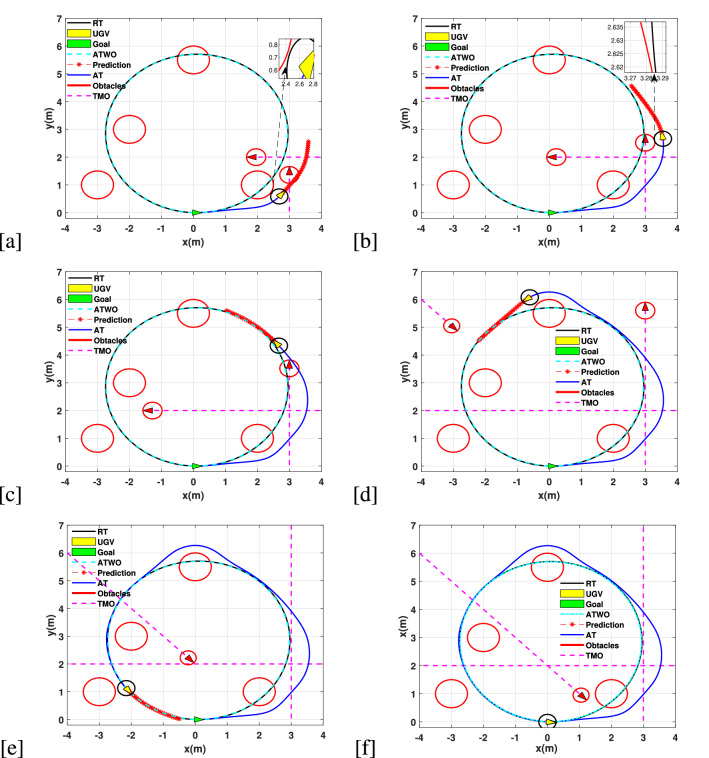
(**a**) Avoided first SO and MO by predicting them. (**b**) Speeds up to safely overtake the incoming vehicle. (**c**) Catching up the RT (**d**) Avoiding another SO and predicting incoming MO to slow down. (**e**) Approaching to the goal location. (**f**) Safely reached to the goal.

**Figure 5:**
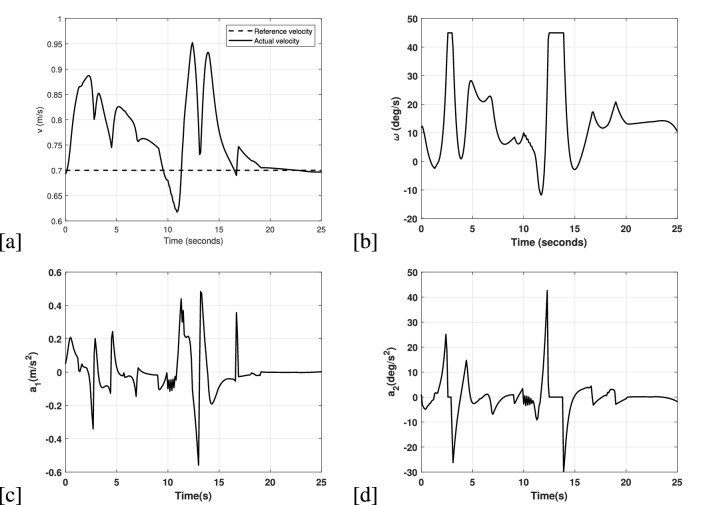
(**a**) Longitudinal velocity applied to the UGV in the simulation experiment 3. (**b**) Angular velocity applied to the UGV in the simulation experiment 3. (**c**) Rate of change in the longitudinal velocity applied to the UGV in the simulation experiment 3. (**d**) Rate of change in the angular velocity applied to the UGV in the in the simulation experiment 3.

**Figure 6:**
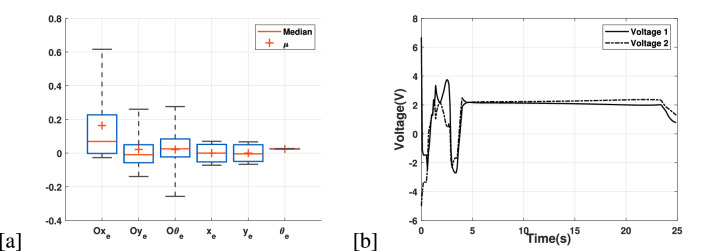
(**a**) Box plot to distinguish the actual state tracking errors; where pose (x,y) are in *c* *m* and heading is in *rad*. Additionally, $Ox_e,\,Oy_e$ and $O\theta _e$ represents obstacles case. (**b**) Applied voltages to the UGV in the simulation experiment 3.

We have considered four SOs and three MOs as shown in the Fig. 4. The SOs are located at $(x_1,\,y_1) = (2,\,1)$, $(x_2,\,y_2) = (0,\,5.5)$, $(x_3,\,y_3) = (-3,\,1)$, and $(x_4,\,y_4) = (-2,\,3)$. All the SOs are having a radius of 0.5*m*. For MOs, the starting locations are $(x_5,\,y_5, \theta ) = (-8,\,10,\, pi/4)$, $(x_6,\,y_6,\theta ) = (3,\,0,\,pi/2)$, and $(x_7,\,y_7) = (4,\,2,\,-pi)$, while these MOs are subject to linear obstacles with linear velocities of $0.5 \, {m/s},\,0.4 \, {m/s}$, and $0.6 \, {m/s}$, respectively. The intention for this simulation is to avoid collisions and follow the reference trajectory. For first collision avoidance, the UGV predicts SO and MO to divert from the reference trajectory as shown in the Fig. 4a. Since, the longitudinal velocity of the vehicle is $1\, {m/s}$, it increases speed to avoid the MO safely as shown in the Fig. 4b. In particular, this is an example of a vehicle over taking another vehicle in the environment. The UGV tracks back on the proposed nominal trajectory, as shown in the Fig. 4c, in which the achieved platform’s trajectory does converge to the nominal trajectory, after a short transient during the collision avoidance event. Soon after synchronizing with the nominal trajectory, the platforms detects the presence of an unexpected SO, which requires another collision avoidance manoeuvre, as shown in Fig. 4d. In Fig. 4e the platform properly continues the trajectory tracking process, for finally dealing with a second incoming DO and an additional SO, both requiring an avoidance treatment, which is successfully performed, for finally completing the required trajectory as shown in Fig. 4f.

**Table 1:** NMPC average *PT*/*I* for single and multi-core CPUs.

${N_{p}}$	Single core this paper (ms)	Single core^26^ (ms)	Multi-core this paper (ms)	Multi-core^26^ (ms)
15	1.2	10.3	1	10
50	3.32	21.47	3	21
100	10.6	38.61	10	38
150	17.8	51.82	17	51

**Table 2:** Comparison of Euclidean distance accuracy among MS and SS numerical methods.

${N_{p}}$	This paper (accuracy (cm))	Accuracy (cm)^26^
15	− 3.0 to 3.0	− 10.0 to 10.0
50	− 0.5 to 0.5	− 2.5 to 2.5
100	− 0.05 to 0.05	− 0.25 to 0.25
150	− 0.01 to 0.01	− 0.01 to 0.01

In terms of meeting the input constraints imposed by the NMPC, Fig. 5 illustrates the applied velocities and accelerations to the UGV. The reference velocity is considered as 0.7*m*/*s*, which the vehicles tries to match. However, since the environment is filled with obstacles, it can only reach the reference velocity once the obstacles are avoided as shown in the Fig. 5a. Additionally, the longitudinal velocity increases in the time instances $10(sec)-15(sec)$, which indicates avoidance of obstacles safely and then converging to the reference velocity. For the angular velocity, a similar trend can be observed in the Fig. 5b. For the rate of change in the longitudinal and angular velocities in Fig. 5c,d, chattering are visible in the signal. However, it always converges to zero once it meets the reference trajectories. The reason for having chattering are due to the continuous occurrence of multiple obstacles, which diverts the UGV from it’s nominal trajectory.

In terms of trajectory tracking error, we have computed a case with no obstacles and compared it with the one, which we have simulated in this experiment as shown in the Fig. 6a. The mean error for both situations are respectable. However, it is appreciable that without obstacles the tracking error is very small, while the error with obstacles are still having reasonable values. Additionally, for control action applied to the UGV, the applied voltages are converging to a steady-state as shown in the Fig. 6b, which proves that motor would work adequately despite these challenging conditions of obstacles and skid-slip.

### Performance evaluation of the proposed method

To validate the robustness of the proposed method, we have evaluated our method with^26^. The comparison is performed as per the error accuracy, capability of the NMPC to run on a single core and multiple cores CPU, and average processing time of the NMPC to compute a single optimization iteration. As mentioned earlier in the paper, our numerical method is based on multiple shooting problem, which is tested in different prediction horizon of time.

The ability to perform any optimization method on an on-board processor must be feasible. For instance, a single-core CPU with limited resources must be able to solve the optimization problem effectively. For this comparison, Table 1 discusses the proposed method utilizes the average processing time per iteration (*PT*/*I*) for this paper (using MS) and compared with SS. In terms of computing NMPC on single or multi-core CPUs, the average *PT*/*I* in this paper is relatively better than SS method. In addition, even if we increase the prediction horizon to a larger number ${N_{p}} = 150$, the *PT*/*I* always feasible to run this on the CPU with limited resources. This also enables to perform a real experiment with an on board processor with limited processing capabilities.

In contrast to^26^, a remarkable finding is that accuracy in the MS method is optimal for a small and a large prediction horizon. Although, an increase in the prediction horizons may cause a slower processing but a lower error as shown in the Table 2. However, the issue with increasing a too large prediction horizon isn’t feasible for some cases. For instance, in^26^, a prediction horizon of ${N_{p}} = 100$ was used to run the path planner, which has an accuracy of 2*cm*. However, this can lead to a larger processing time. Thus, based on these findings, the proposed method is reasonable for real-time applications.

### Skid-slip analysis

**Figure 7:**
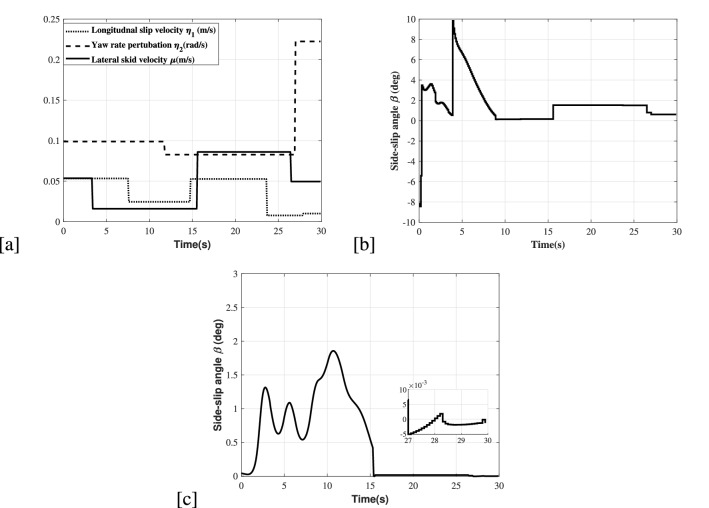
(**a**) PWL function-based slippage and skidding velocities. (**b**) Side slip angle of the front wheels for the simulation 1. (**c**) Side slip angle of the front wheels for the simulation 2.

In terms of including the skid-slip applied to simulations, we have also used the PWL-based function proposed in Fig. 7a for the circle trajectory simulation. Furthermore, the side-slip angle of front wheels is within a reasonable range (slip angle) $\beta \le |10deg|$ as shown in Fig. 7b,c.

## Conclusions

In this paper, we propose a tracking control and planner for a UGV in the presence of a skid-slip and dynamic and static objects. The proposed technique consists of a fast NMPC-based path-planner, novel evolutionary programming-based kinematic controller, a dynamic controller with a nonlinear disturbance observer, and a voltage driving circuit. We have performed simulation experiments by considering actual physical parameters to drive the vehicle safely to the goal location. First, a large map of UNSW is used, which is fed to PPQ-Dijkstra algorithm, and NMPC is combined with the map to avoid collisions and non-holonomic constraints. Finally, a test in which the UGV must follow a sizeable circular trajectory under the presence of static and moving objects is performed. The results obtained from these experiments validate that the proposed method can safely drive the vehicle to the goal location. To validate the performance of our proposed technique, we have performed the numerical multiple-shooting method, being restricted to use only one core and running in multiple cores. Compared to the existing literature, the proposed technique has improved the processing capability of the NMPC to run in a CPU with limited resources and improves the error accuracy.

## List of symbols

$$a_r$$: Reference Acceleration
$q$: Actual trajectory of the UGV
$q_r$: Reference trajectory of the UGV
$q_g$: Initial states of the UGV
$S(q)$: Kinematics function of UGV
$\Omega$: actual velocity vector
$\Omega _r$: Proposed velocity vector
$\eta$: Perturbed angular velocities due to slipping of the two actuated wheels
$\rho$: Mismatched disturbances vector
$\mu$: Lateral skidding velocity of the UGV
$M$: Symmetric and positive-definite inertia matrix
$H$: Centripetal and coriolis matrix
$B$: Input transformation matrix
$\eta _1$: Longitudinal slip velocity
$\eta _2$: Yaw rate perturbation due to slippage of the wheels
$v$: Longitudinal velocity of the UGV
$\omega$: Yaw rate of the UGV
$\tau$: Applied torque vector
$\tau _d$: Torque disturbance vector
$r$: Radius of wheels
$m$: Mass of the UGV
$d$: Distance between the centre of mass of the UGV and wheel axis
$\theta$: Heading of the UGV
$x,\,y$: Position of the UGV
$J$: Moment of inertia
$N$: Mechanical gear ratio
$$N_p$$: Prediction horizon
$V_u$: Applied DC voltage to the motors
$$L_a$$: Rotor inductance
$$R_a$$: Rotor resistance
$$K_t$$: Torque constant
$$i_a$$: Armature current
$$T_s$$: Sampling time
$$F_s$$: Sampling frequency
$$k$$: Discrete indexing
$$C_T$$: Terminal cost
$$C_S$$: Stage cost
$Q$: Semi-positive state-penalty gain matrix
$R$: Diagonal positive definite input-penalty gain matrix
$\bar{Q}$: Semi-positive terminal cost state-penalty gain matrix
$q_e$: State tracking error matrix
$u_c$: compensated velocity vector
$u_p$: Tuned velocity vector
$$F$$: Dynamic matrix
$$T$$: Error matrix
$\zeta$: Velocity tracking error
$\Delta \tau$: Rate of change in torque vector
$L$: Observer gain matrix
$$\hat{D}$$: Auxiliary variable
$K$: Constant matrix to design observer
$\alpha$: Diagonal positive definite matrix
$\beta$: Controller gain matrix
$K_c$: Input saturation compensation gain
$r_c$: Radius of the bounding circles of UGV
$r_o$: Radii of the obstacles
$k_x$: Kinematic compensation gain for x
$k_y$: Kinematic compensation gain for y
$k_\theta$: Kinematic compensation gain for $\theta$
$\hat{k_x}$: sub-optimal scaling factor for x
$\hat{k_y}$: sub-optimal scaling factor for y
$\hat{k_\theta }$: sub-optimal scaling factor for $\theta$
$K_f$: Electromotive force coefficient
$K_c$: Input saturation’s compensation gain
$$W$$: Evolutionary weights
$$P$$: Population vector
$S(q)$: Kinematics function of UGV
$C$: Centripetal and coriolis matrix
$C_{opt}$: Optimal individuals of population P of evolutionary programming
